# SAPHO syndrome with pathological fractures of vertebral bodies: a case report

**DOI:** 10.1186/s12891-019-2410-x

**Published:** 2019-01-17

**Authors:** Yalong Li, Guomin Liu, Yian Zhao, Yungang Luo, Tiancheng Lu

**Affiliations:** 1 Department of Orthopedics, the Second Hospital of Jilin University, Changchun, Jilin, 130041 People’s Republic of China; 2 Department of Stomatology, the Second Hospital of Jilin University, Changchun, Jilin, 130041 People’s Republic of China; 3 Life Sciences College, Jilin Agricultural University, Changchun, Jilin, 130118 People’s Republic of China; 4 Jilin provincial Changbai mountain medicine anti-tumor engineering center, Jilin, People’s Republic of China

**Keywords:** SAPHO syndrome, Bisphosphonates, Osteomyelitis, Steroids, Multiple bone lesions

## Abstract

**Background:**

It’s difficult to diagnose and treat synovitis-acne-pustulosis-hyperostosis-osteomyelitis (SAPHO) syndrome due to its rare and unknown pathogenesis. There is no effective treatment for SAPHO syndrome and the consequences of empirical treatment are unpredictable. This study reports a case of a young female diagnosed as SAPHO syndrome with pathological fractures of vertebral bodies.

**Case presentation:**

A 29-year-old female complained of the right sternoclavicular joint and back pain accompanied limited activities and cutaneous lesions. Laboratory assays revealed abnormal inflammatory factors. Multiple imaging studies illustrated bone lesions and pathological fractures of vertebral bodies. A diagnosis of SAPHO syndrome was made. The patient was treated with Compound Troxerutin and Poreine Cerebroside Injection, non-steroidal anti-inflammatory drugs (NSAIDs), bisphosphonates, corticosteroids and the thoracolumbar brace. The patient was followed up for 6 months and showed improved results.

**Conclusions:**

The case supports that multiple image inspections and laboratory tests contribute to diagnose SAPHO syndrome, and combination therapies of Compound Troxerutin and Poreine Cerebroside Injection, NSAIDs, bisphosphonates, corticosteroids and the thoracolumbar brace in the treatment of SAPHO syndrome with pathological fractures of vertebral bodies are crucial to regain health.

## Background

SAPHO syndrome, an acronym for synovitis-acne-pustulosis-hyperostosis-osteomyelitis, is a rare disease with an estimated prevalence of 0.00144/100000 [1]. SAPHO syndrome was first introduced by Chamot in 1987 [2] and characterized by both cutaneous lesions and osteoarticular manifestations [3]. Besides, the latest study demonstrated that it can result in symptoms of depression [4]. There are few case reports which may be attributed to its extremely low prevalence and difficulty to diagnose or missed diagnosis. This study reports a case of young female diagnosed as SAPHO syndrome with pathological fractures of vertebral bodies.

### Case presentation

A 29-year-old female complained of the right sternoclavicular joint and back pain accompanied limited activities and pustulosis-like rashes on the palms for 1 month without any clear predisposing cause. She took analgesic medicine by herself (an unknown analgesic), while without obvious effect. Physical examinations on admission revealed pustules on the palms and multi-erythematous nodules on the lower legs. There were also redness, swelling and tenderness in the right sternoclavicular joint area and tenderness in the lower back.

Laboratory assays revealed an elevation of the erythrocyte sedimentation rate (ESR, 87 mm/h, normal range 0-20 mm/h), levels of C-reactive protein (CRP, 28.30 mg/L, normal range 0–7.44 mg/L), prothrombin time (12.7 s, normal range 9.4–12.5 s), fibrinogen assay (5.13 g/L, normal range 2-4 g/L) and complement C4(40 mg/dL, normal range 16-38 mg/dL), and a slightly decline of hematocrit (33.9%, normal range 35–45%). In addition, rheumatoid factor(RF) and human leukocyte antigen B27(HLA-B27) tests were negative. The results for the remainder of her biochemistry and hematology were within normal range, including immunoglobulins, antinuclear antibody (ANA) spectrum and tumor markers.

**Fig. 1:**
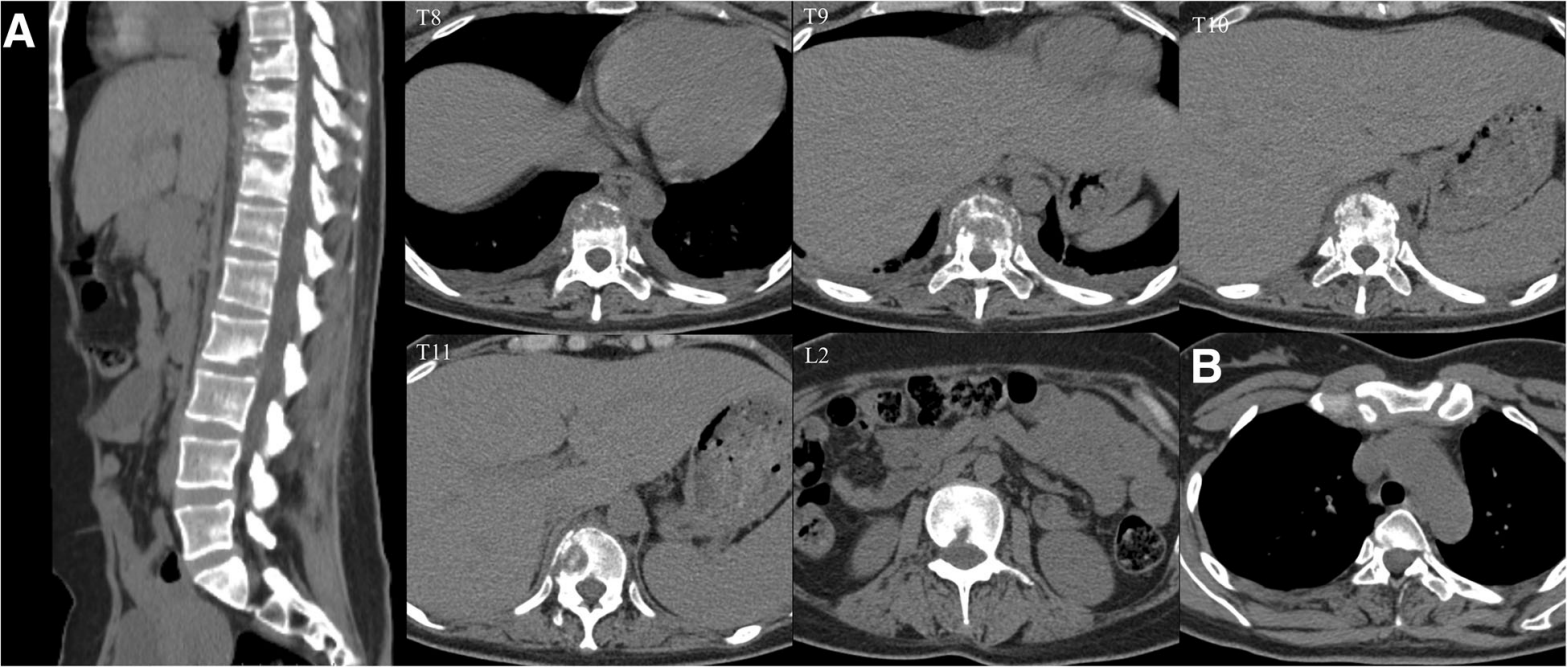
CT scans of the thoracic and lumbar spine revealed erosions, hyperostosis, and osteosclerosis in the T8–11 and L2 vertebral bodies and fractures in the T8–11 vertebral bodies (**a**). The cortical bone of the sternum and sternoclavicular joint was continuous without damage (**b**)

**Fig. 2:**
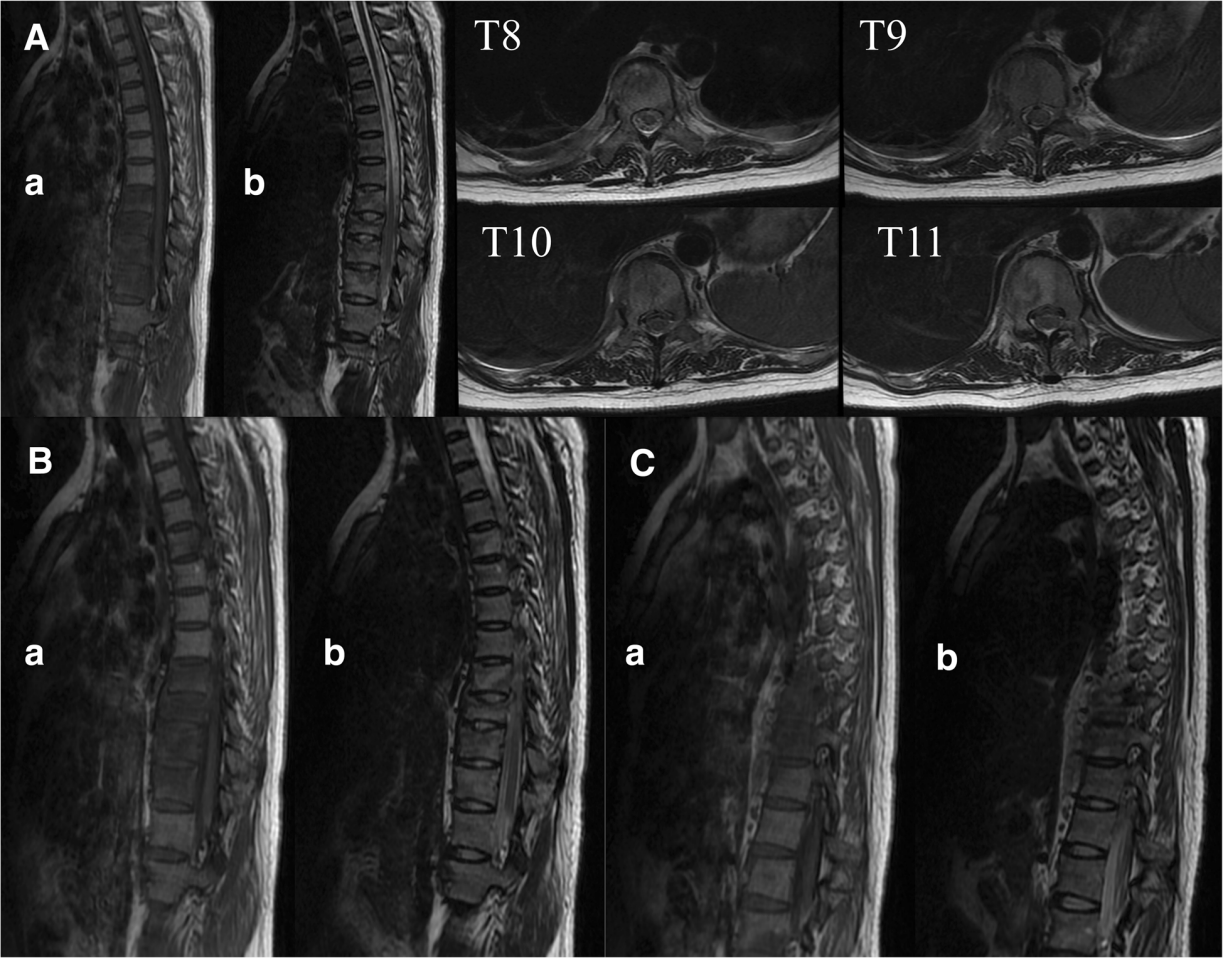
MRI scans of the thoracic showed superior end plate compression deformity in the T4 (**b**) and T8–11 vertebral bodies (**a**). In addition, diffused inflammatory bone changes were also noted in the T4 (**b**), T8–11(**a**) and L2 (**c**) vertebral bodies that were hypointense on the T1-weighted image (**a**) and hyperintense on the T2-weighted image (**b**)

**Fig. 3:**
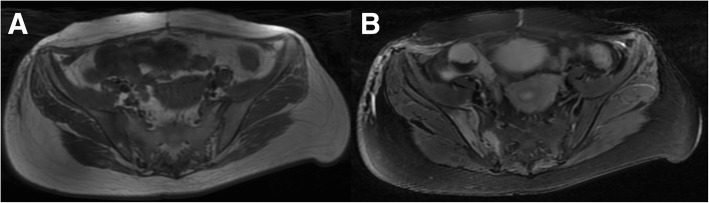
Axial MRI scans of the sacroiliac joints illustrated inflammatory bone changes at the right sacroiliac joint that were hypointense on the T1-weighted image (**a**) and hyperintense on the T2-weighted image (**b**)

**Fig. 4:**
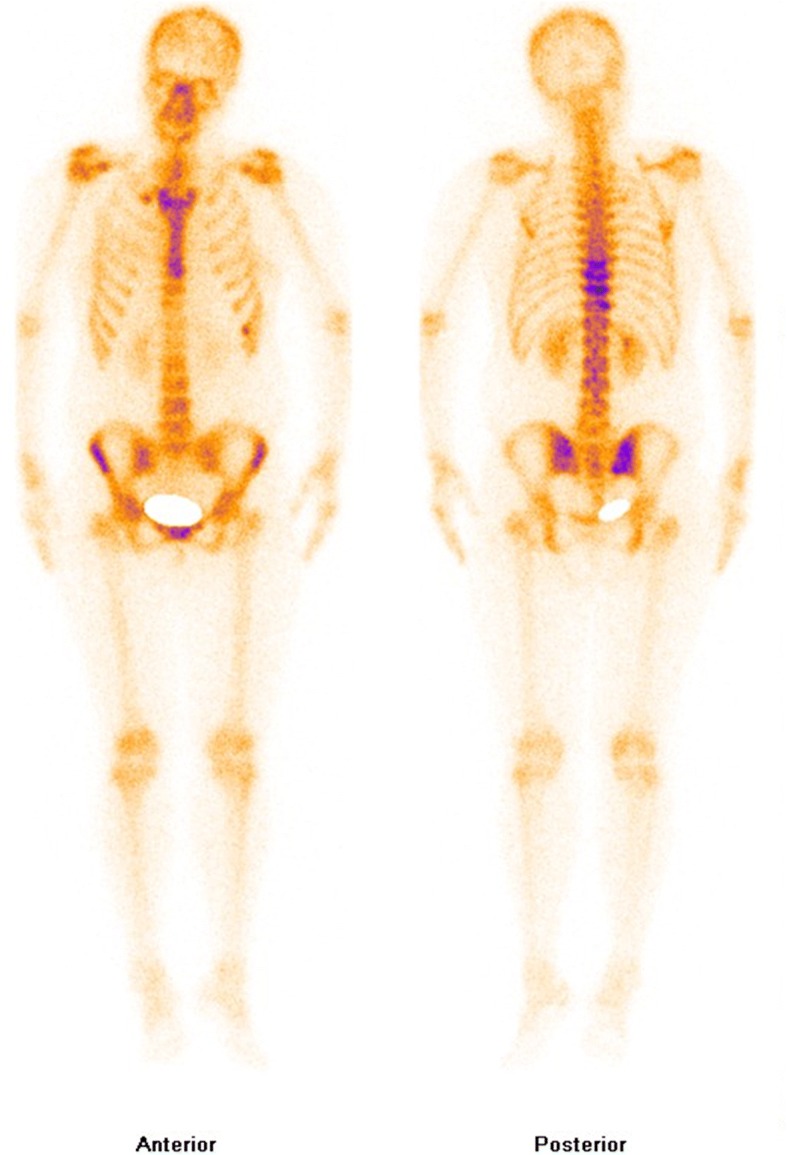
The WBS showed diffuse intense uptake at the proximal end of the right clavicle and seventh anterior rib, manubrium sterni, right sacroiliac joint, pubic symphysis and in the T8–11 thoracic vertebral bodies duo to inflammatory bone changes

**Fig. 5:**
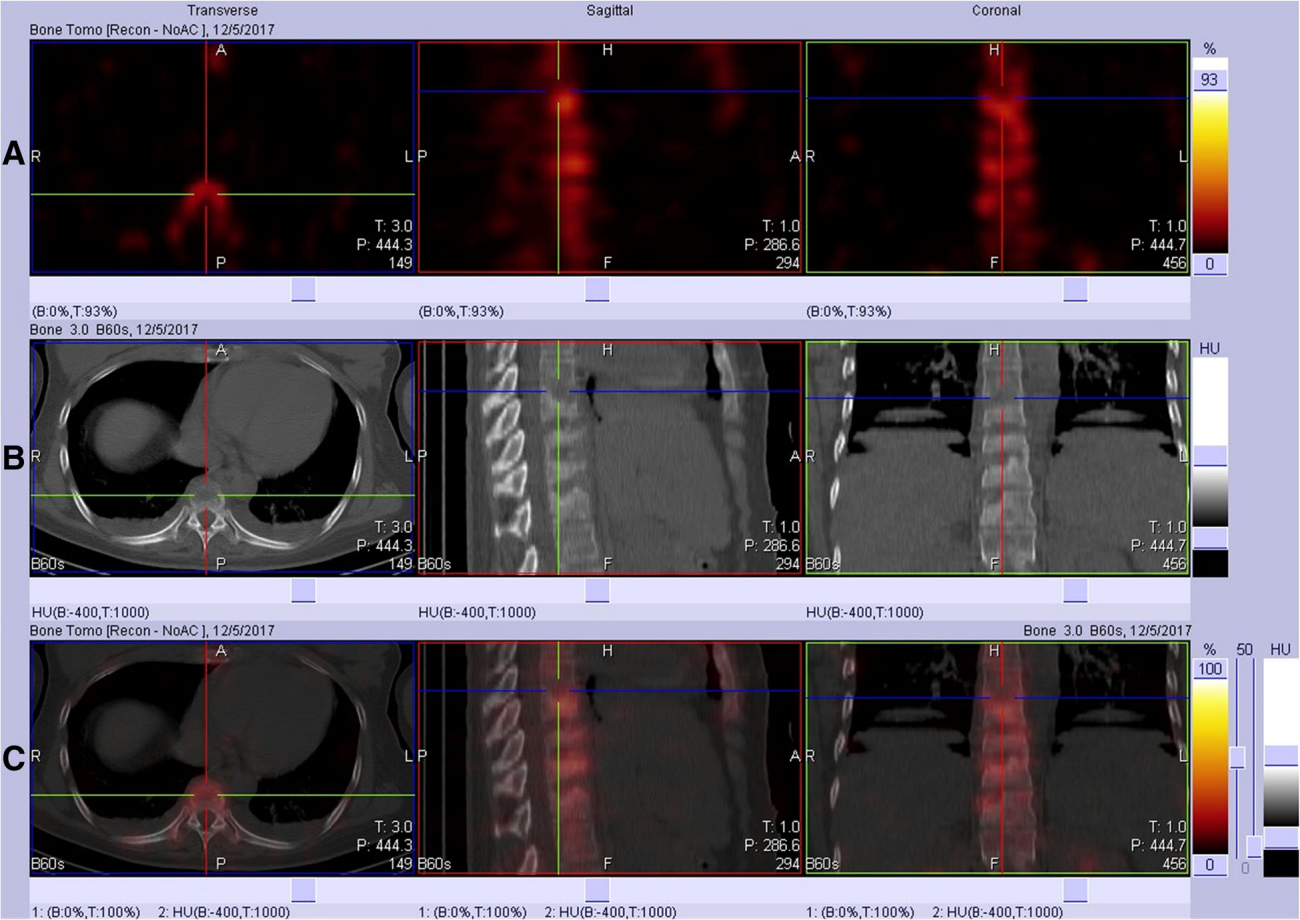
SPECT/CT imaging of thoracic and lumbar revealed marginal erosions and superior endplate compression deformity in the T8–11 vertebral bodies. **a**: SPECT imaging; **b**: CT imaging; **c**: Fusion imaging

**Fig. 6:**
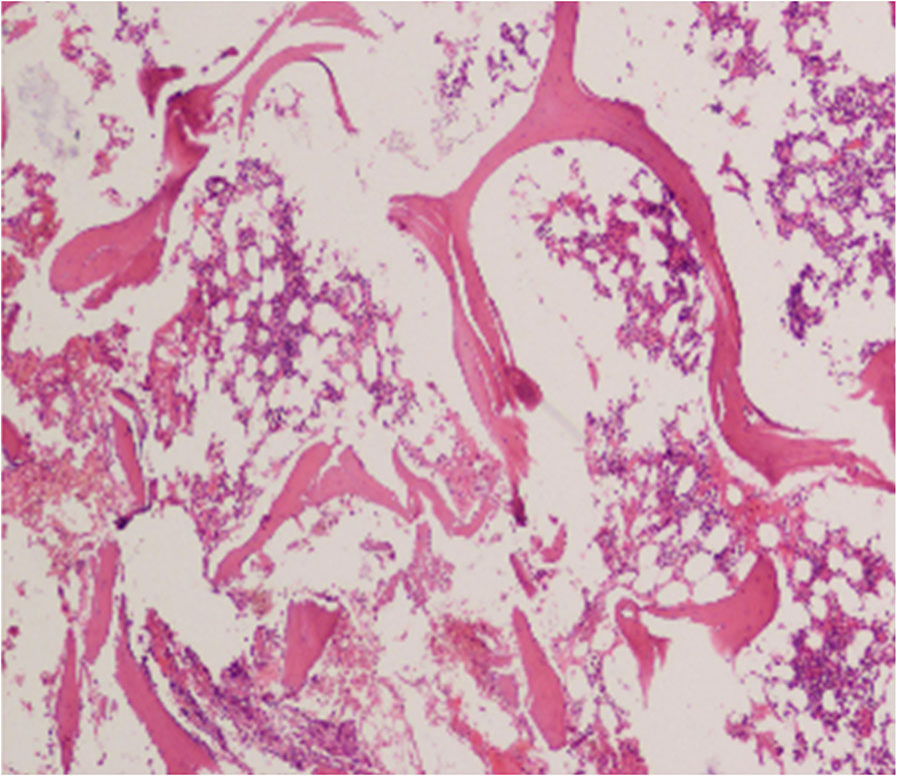
Pathological section of iliac bone lesions illustrated neutrophil-dominant osteitis (H&E, 400×)

Computerized tomography (CT) scans of the thoracic(T) and lumbar spine revealed multiple vertebral lesions (T8–11 and T2 vertebral bodies) while without the sternum and sternoclavicular joints (Fig. 1). Magnetic resonance imaging (MRI) scans of thoracic spine and ankle demonstrated multiple vertebral lesions (T4, T8–11 and L2 vertebral bodies), right ankle arthritis and pathological fractures (T9–10 vertebral bodies) (Fig. 2 and Fig. 3), same as CT scans. We diagnosed the patient with SAPHO syndrome. For further diagnosis, a whole body bone scan (WBS) was performed 4 h following the injection of 20 mCi ^99m^Tc-methylene-diphosphonate(Fig. 4). Anterior and posterior views of the WBS illustrated intense uptake at the proximal end of the right clavicle, left first front rib, T8–11 vertebral bodies, right ankle joint and pubic symphysis. The thoracolumbar Single-Photon Emission Computed Tomography (SPECT)/CT fusion imaging showed a low density of bone in the same location and different levels of radioactivity uptake around the lesioned bone (Fig. 5). Pathological section of tibial lesions demonstrated massive neutrophil infiltration in bone marrow(Fig. 6).

A diagnosis of SAPHO syndrome was made. For the vertebral fractures, the patient was treated conservatively with the thoracolumbar brace and bed rest for 3 months. Besides, the patient was treated with dynastat (80 mg, BID) until the pain eased or disappeared and Compound Troxerutin and Poreine Cerebroside Injection(10 ml, BID)for improving blood circulation on the fourth and tenth days. And Technetium [^99^Tc] Methylenediphosphonate Injection (^99^Tc-MDP, Product Name: Yunke Injection, 3 sets, QD)in combination with betamethasone sodium phosphate injection (20 mg, QD) was introduced on the tenth day for treating SAPHO syndrome. The patient experienced a moderate decrease in the intensity of the pain without any anodyne. On the seventeenth day, betamethasone sodium phosphate injection (12 mg, QD) was introduced again. There were normal ESR (8 mm/h) and CRP (0.36 ng/L), improved dermatoses and no relapse of joint pain on the eighteenth day. The patient was discharged on the twentieth day. During the 6-month telephone follow-up of the patient (phones are followed up every half month), there was no relapse of joint pain according to the patient’s description, however, the patient refused to review for economic reasons.

## Discussion

Chamot et al. [2] first proposed the SAPHO syndrome and the initial diagnostic criteria in 1987. SAPHO syndrome is at present considered a rare disease and sufficient data on its prevalence are unavailable, while for the lack of correct diagnosis, its actual prevalence may be underestimated. SAPHO syndrome has a female predominance among patients [5, 6], especially younger than 30 years old [5]. Despite all of this, it is considered that SAPHO syndrome may present at any age [5–7], even in an only 15-month-old childhood [8]. The etiopathogenetic mechanism of SAPHO syndrome, although having been proposed involving bacteriologic, immunologic and genetic factors [3] such as autophagy, interleukin-1(IL-1), Forkhead Box O1(FoxO1) and propionibacterium (Cutibacterium) acnes [9], remains poorly understood.

The most frequently mentioned diagnostic criteria for SAPHO syndrome was proposed by Kahn and Khan [10] in 1994 (Table.1), and the more precise diagnostic criteria was modified by Kahn in 2003 [11] (Table.2). It’s difficult, however, to make a diagnosis in certain case, such as no skin lesions [12]. Not only the history and clinical signs play a crucial role in diagnosis, but also imaging findings and laboratory tests. Some studies have demonstrated that erythrocyte sedimentation rate and CRP may be elevated [13] for SAPHO syndrome, and our report also confirmed this. Besides, we had not detected any autoantibodies, including RF, HLA-B27, immunoglobulin and ANA spectrum.

**Table 1:** Diagnostic criteria proposed by Kahn for SAPHO syndrome diagnosis, 1994 [10]

1. Chronic recurrent multifocal sterile and axial osteomyelitis, with or without dermatosis	
2. Acute, subacute, or chronic arthritis associated with palmoplantar pustulosis, pustulous psoriasis, or severe acne	
3. Any sterile osteitis associated with palmoplantar pustulosis, pustulous psoriasis, or severe acne

**Table 2:** Diagnostic criteria proposed by Kahn for SAPHO syndrome diagnosis, 2003 [11]

Inclusion criteria:	
Bone–joint involvement associated with palmoplantar pustulosis and psoriasis vulgaris	
Bone–joint involvement associated with severe acne	
Isolated sterile* hyperostosis/osteitis (adults)	
Chronic recurrent multifocal osteomyelitis (children)	
Bone–joint involvement associated with chronic bowel diseases	
Exclusion criteria:	
Infectious osteitis	
Tumoral conditions of the bone	
Non-inflammatory condensing lesions of the bone	

*Exception: growth of Propionibacterium acnes

Note: Table 1 and Table 2 should appear at the second paragraph of the ‘Discussion’ section

A “bull’s head” appearance shown in bone scintigraphy is the characteristic of SAPHO syndrome [10],while bone scintigraphy in our case only revealed intense uptake at the proximal end of the right clavicle and manubrium sterni, which may be related to the patient was in the early stage of the disease. What’s more, SAPHO syndrome is heterogeneous, different patients may have different performance. In adult patients, the most frequent area are the anterior chest wall, accounting for 60–95%, especially in the costochondral, sternoclavicular, manubriosternal and costosternal junctions, and spine, accounting for 32–52%, most commonly in the thoracolumbar spine [14]. The radiological results of SAPHO syndrome comprise hyperostosis, osteolysis, osteitis, and osteosclerosis [3]. In this report, CT scans, MRI scans and SPECT/CT fusion image demonstrated a detailed image of multiple bone destructions, vertebral fractures and sclerosis formation surrounding lesion bones. For the patient, the complaint of back pain and limited activities were due to vertebral fractures, which was resulted from osteolysis of T8–11 vertebral bodies. And the latest research showed that ultrasound can also contribute to the diagnosis of SAPHO syndrome [15]. For excluding malignancy, bone biopsy was performed. Infiltration of large numbers of neutrophils supports osteitis.

As far as we know, there is no effective treatment to cure SAPHO syndrome. Current treatment of this illness is mainly focused on relieving symptoms. NSAIDs are the first-line treatment. Several studies have supported the effectiveness of bisphosphonates, steroids, methotrexate, tumor necrosis factor (TNF) inhibitors and so on as effective treatments [16–18]. While before we treated this patient, several reports had shown that some patients may develop or have paradoxical worsening of skin lesions after starting TNF inhibitors with unclear reasons [19, 20], which was confirmed by a recent array research [21]. In this study, conservative treatments including Compound Troxerutin and Poreine Cerebroside Injection, NSAIDs, bisphosphonates, corticosteroids and the thoracolumbar brace were applied to treat SAPHO syndrome and pathological fractures of vertebral bodies resulting from it, and the combination proved to be effective for this disease according to the observation results of the patient.

## Conclusion

According to our study, multiple image examinations and laboratory tests are helpful for the diagnosis of SAPHO syndrome, and combination therapies of Compound Troxerutin and Poreine Cerebroside Injection, NSAIDs, bisphosphonates, corticosteroids and the thoracolumbar brace in the treatment of SAPHO with pathological fractures of vertebral bodies are vital for controlling the disease.

## Abbreviations

ANA: Antinuclear antibody
BID: Twice a day
CRP: C-reactive protein
CT: Computerized tomography
FoxO1: Forkhead Box O1
IL-1: Interleukin-1
MRI: Magnetic resonance imaging
NSAIDs: Non-steroidal anti-inflammatory drugs
QD: Once a day
RF: Rheumatoid factor
SAPHO: Synovitis-acne-pustulosis-hyperostosis-osteomyelitis
SPECT/CT: Single-Photon Emission Computed Tomography
T: Thoracic
TNF: Tumor necrosis factor
WBS: Whole body bone scan
